# Settling secondhand sales: Pricing symbolic items in an emergent online marketplace environment

**DOI:** 10.1111/1468-4446.13168

**Published:** 2024-11-17

**Authors:** Ryan Fajardo

**Affiliations:** ^1^ Department of Sociology Northwestern University Evanston Illinois USA

**Keywords:** economic sociology, markets, platforms, prices, valuation

## Abstract

How do sellers on online marketplaces determine agreeable prices? This question is a theoretical concern for sociologists but a professional one for secondhand clothing resellers. Thousands of resellers across the United States purchase items from physical secondhand clothing sources and then resell them for a profit on sites such as Depop, Etsy, and Poshmark. They confront two pricing challenges: secondhand clothing items are aesthetic items of non‐standard, uncertain quality, and online marketplaces offer limited explicit institutional support to back pricing claims. I analyze interviews and fieldwork to theorize how resellers price items for sale on online marketplaces. Resellers gain knowledge of secondhand community values and online marketplace technologies via immersion into offline (local reselling networks and secondhand sources) and online spaces (social media and the marketplaces themselves). Resellers selectively draw on these sources of pricing knowledge to deploy similar but varied pricing practices. These situated valuation practices reveal how resellers rely on reselling community structures and reflexively invoke pricing displays on marketplace interfaces to price secondhand clothing. These practices increase confidence in exchange as resellers can suitably justify the prices of material goods to online marketplace participants with varying levels of knowledge and experience.

## 1 ‬‬‬‬INTRODUCTION

How online marketplaces achieve agreeable prices is an open question within economic sociology. Pricing–valuing and monetarily quantifying material products or services–are central market concerns (Beckert, 2009‬‬: 247). Pricing practices are uniquely uncertain in online marketplaces, organized virtual spaces where trade occurs through digital platforms (Aspers & Darr, 2022, p. 824). Online market participants face high uncertainty because participants lack familiarity and trust, competition is high, and demand constantly fluctuates (Kirchner & Schüßler, 2019, p. 132). To help regulate these uncertainties, online marketplace managers introduce technical interfaces such as public displays of past and present prices. They also incorporate varied pricing formats, which differ in the degree of formal control, from user‐negotiated pricing to structured auctions and automatic algorithmic computation (Rilinger, 2024, p. 6–9). Subsequently, it is unclear how online marketplace participants navigate these heterogeneous pricing features and overcome market uncertainty.‬‬‬‬‬‬‬‬‬‬‬‬‬‬‬‬‬‬‬‬‬‬‬‬‬

The emergence of online marketplaces has changed pricing in one trade area: secondhand clothing (i.e., clothing resold after an initial sale). Market actors historically valued secondhand clothing at bargain prices. For over a century, most secondhand clothing trade in the United States (US) occurred in offline charity outlets or marginalized commercial venues like flea markets (Le Zotte, 2017).^1^ Over the last decade, online marketplaces captured considerable portions of the secondhand clothing trade. According to industry reports, marketplaces like Depop, Etsy, Poshmark, and thredUp now command a 41.7% ($15 billion) share of all secondhand clothing trade in the US (thredUP, 2024).^2^ Thousands of independent resellers across the US and Europe drive this transformation. They trawl through physical secondhand clothing sources to locate and purchase used, ready‐to‐wear clothing items offline.^3^ They then perform arbitrage by reselling these items online at higher prices.

Many secondhand clothing items are of uncertain quality due to differences in physical condition and disconnect from past owners (e.g., de la Pradelle, 2006, p. 169; Norris, 2012, p. 131). Although economic sociologists describe secondhand clothing trade as value‐laden (Fischer, 2015), the underlying pricing processes of resellers remain ambiguous. Unlike bookers and clients in high fashion (c.f. Mears, 2011, pp. 128, 139) and participants in similar symbolic or aesthetic industries, resellers receive little training and often only associate with other resellers loosely. Furthermore, the industry is not ‘densely institutionalized’ like the worlds of wine (Hay, 2010, p. 686) or fine art (Velthuis, 2005), which feature critical and expert analysis, trade publications, and professional groups. Rather, resellers determine prices of uncertain, symbolic goods with new tools, mixed organizational support, and little formal advice.

How do resellers agreeably price secondhand clothing items for eventual sale online? Using interviews and fieldwork, I show how pricing practices among a sample of online secondhand clothing resellers converge through their immersion into offline (local reselling networks and secondhand sources) and online spaces (social media and the marketplaces themselves). Resellers construct lay pricing scripts and heuristics based on signifiers prized in the secondhand community. Concurrently, they champion online marketplace pricing displays for providing access to ‘true’ market values, which they reflexively invoke to support valuations. Resellers thus do ‘performative work,’ integrating online marketplace pricing devices into market practice while attending to contextual social norms, roles, and values (Beunza & Ferraro, 2019, pp. 535–36). Prices online thus enable ‘the mutual constitution of taken‐for‐granted knowledge’ of a perceived true market value for clothing and ‘institutionalized structures among market actors’ that guide how resellers discern value differences (Bååth, 2023, p. 7). Through these practices, resellers effectively improve confidence in and legitimate prices to both new and experienced–often anonymous–users (Beckert, 2020, p. 292; Kirchner & Schüßler, 2019, p. 132; Wherry, 2008).

## 2 PRICING IN ONLINE, SYMBOLIC MARKETPLACES

Prices are the product of pricing and a source of market knowledge itself. Pricing is a form of valuation or ‘assessing, of ascribing, and appreciating values’ (Bååth, 2022, p. 63). Through pricing, actors translate certain cultural and material values identifiable in items and services into economic prices (Çalışkan, 2007, p. 257). Prices also serve as practical, everyday market knowledge actors use to arrange the market. Market participants compare and evaluate competing offers using prices (Beckert, 2011, p. 2), and price differentiation can signal differences in quality and desirability (Bååth, 2023, p. 7).

Economic sociologists offer two seemingly competing theories to explain pricing processes, though they are often siloed in empirical research.

Structural theories detail how formal (e.g., governmental regulation) and informal (e.g., local cultural values) social structures regulate pricing (Beckert, 2020). Close friend networks, third‐party rating agencies, or professional associations define pricing practices within a market context (Bååth, 2023, pp. 8–9). These institutional contexts circulate cultural norms, which often materialize into formal rules or guidelines, such as safety standards, rating scores, and seals of approval (Beckert, 2011, pp. 18–19). Market participants often incorporate these components into scripts, or cognitive frames that guide behavior, to ground pricing expectations over time and across groups (Velthuis, 2005, p. 117). From this structuralist view, coordinative processes across and within social structures determine pricing practices.

Alternatively, performative theories show that how central actors think markets work, including pricing, is not a matter of description but fundamental construction (e.g., Callon, 2016; MacKenzie et al., 2007; Muniesa, 2007). Central actors arrange markets to encourage certain behaviors and delimit others, shaping economic power and resource distribution. From this view, the material and cognitive construction of markets is not value‐neutral but reflects purposeful decisions informed by expertise and a desire to influence exchanges (Callon & Çalışkan, 2009, p. 3). Consequently, performativity scholars view material aspects of price, such as its display, ability to move, and how it moves, as market devices that give form to collaborative economic action (Cochoy et al., 2021, p. 135). These market devices structure action globally. They allow disparately located and isolated market actors to ‘perceive the market,’ ‘contribut[ing] to the ways in which [they] carry out calculations and plan their trading practices’ (Çalışkan, 2009, p. 241).

Existing pricing studies often focus on offline markets. Offline markets refer to direct, in‐person trading environments in physical stores and local marketplaces. In these settings, buyers can inspect material items physically and often negotiate with sellers face‐to‐face. Offline markets are limited in geographic range, as buyers and sellers must be nearby. In contrast, online marketplace trading widens the geographic scope of possible interactions, shifts the nature of identification between traders, alters the possible inspections buyers can perform, and allows market managers to structure and surveil interactions. However, most online marketplaces do not solely feature trade online, and studies on trade in alternative foods (Bååth, 2022) and craft products (Aspers & Darr, 2022) show how online and offline dynamics affect trade in both spheres.

Online marketplaces offer an exemplary opportunity to demonstrate the analytic utility of integrating structural and performative pricing theories for two notable reasons. First, market managers, coders, and UX designers organize marketplaces using technical ‘mechanisms of coordination and social order’ to a greater degree than in offline markets (Kirchner & Schüßler, 2019, p. 143). Online management companies build web interfaces, regulate user interaction, and promote exchange through ‘nudging’ (Rilinger, 2023, p. 886). However, management companies do not solely determine the marketplace environment (Ahrne et al., 2015, pp. 16–17), and sellers' trading practices bring life to markets (Aspers & Darr, 2022). Therefore, it is unclear what principally determines price: marketplace technologies or seller communities.

Second, buyers and sellers online are geographically dispersed and often lack pre‐existing social ties (Kirchner & Schüßler, 2019, p. 132). Participants are often anonymous and repeated interactions are less likely. Many online marketplaces also encourage greater public participation: In online book publishing, the nature of online commerce allows new entrants with varying levels of experience and knowledge into a previously closed field (Fürst, 2019). Since ‘buyers are only willing to engage in market transactions if they can establish the quality of the products offered’ (Beckert, 2020, p. 298), pricing disagreements may occur when individuals remain unknown or differ in their knowledge or experience within the market (Wherry, 2008). In turn, users often rely on digital platform infrastructure to account for confidence issues regarding the quality of goods. Rating and review histories validate prices and increase sales and are central to the legitimacy of sellers' claims (Duxbury & Haynie, 2023). Yet, it is unclear what technologies or practices sufficiently legitimize agreeable prices and inspire confidence in buyers.

These online marketplace dynamics exacerbate pre‐existing challenges of pricing symbolic goods—such as fashion, wine, and art—which are unique and defy immediate comparison (Karpik, 2010). Marketplace actors value these objects for their symbolic, rather than solely their inherent material, worth (Beckert, 2020). The value–and by extension price–of these items results from social rather than material quality assessments (Beckert, 2020, p. 286). In these assessments, sellers attempt to reduce quality uncertainty, inspire confidence from customers for eventual sale, and mobilize against possible critique (Beckert & Rössel, 2013; Boltanski & Esquerre, 2020).

How individual actors assess the quality and price of symbolic products can illuminate general processes of market exchange work. We can look to exemplary studies of fashion. Entwistle (2002) demonstrates that social processes, relationships, and networks among fashion models and bookers establish the former's economic value. Similarly, Mears (2011) describes how model bookers use their metaphoric ‘eye’—shaped by contextual knowledge of widely held modeling industry values—to valuate fashion models (95). These studies reveal how valuation practices are ‘intersubjective processes that unfold between market participants’ (Beckert, 2020, p. 286) resulting from market interaction and reflecting the market orders from which they arise.

This article analyzes the pricing practices of participants in the secondhand clothing market, a symbolic market that is moving from offline to online. I apply insights from structural and performative theories to interviews with online secondhand clothing resellers (Bååth, 2023; Beunza & Ferraro, 2019). In doing so, I attend to secondhand traders' cultural and social context while detailing the role of novel market technologies that express and display prices. This integrative analytic approach reveals how new market technologies influence exchange while resellers maintain considerable pricing control. In this context, prices are outcomes of social processes and market devices that structure future action. Resellers utilize their pricing practices to overcome legitimacy challenges related to popular participation in online marketplaces.

## 3 METHODS AND SAMPLE

This article uses qualitative data I collected in the United States from June 2022 to March 2023 on the secondhand clothing market. I primarily interacted with those who explicitly buy and resell secondhand clothes online for profit. I distinguish resellers from those who only sell their own used clothes. This difference in sourcing methods implies variation in dedication and experience. More so than casual sellers, resellers consistently locate, prepare, and price secondhand clothes. Furthermore, resellers are central to online secondhand clothing marketplaces: They attract a large share of sales and maintain close connections with marketplace managers (Depop, 2023).

I screened potential interview participants by the number of completed online sales (50 sales on any popular secondhand online marketplace). After making initial contact in person or through digital messaging, I verified that individuals actively searched for and resold clothing items.

My recruitment procedures changed throughout my fieldwork. I initially focused on resellers operating in one popular marketplace: Depop. During this initial phase, I realized those operating on Depop used multiple platforms and that limiting recruitment to one marketplace arbitrarily bounded my analysis. I thus expanded my recruitment criteria to include resellers in multiple marketplaces. First, I recruited a convenience sample through contacts I made at in‐person secondhand clothing sale events in Chicago and Los Angeles. Before contacting these resellers, I checked their online presence and ensured they met my sales screening criteria. Second, to determine if pricing practices differed by geographic location, I messaged individuals in the US operating on Depop and Etsy, two popular secondhand clothing marketplaces. I recruited individuals by navigating to popular item pages, such as for ‘shirts’ or ‘pants’, on these two sites and messaging every fifth reseller who satisfied my screening criteria.

I conducted interviews with 31 resellers, 11 recruited from physical markets and 20 from online solicitation. Geographically, interviewed resellers lived all over the US, from Oregon to New York. They typically began reselling in physical isolation, hearing about the work through digital ads or word‐of‐mouth. Only a few had experience working in retail fashion. Resellers held varied work intentions. Some (17/31 resellers) viewed reselling as a side project to secure supplemental income; others (14/31) spent all their working time reselling, up to 60 h a week, potentially earning several thousand dollars each month. Most participants began reselling during periods of personal economic uncertainty, with a third (11 resellers) mentioning employment issues during the COVID pandemic.^4^

I also interviewed nonprofit thrift store managers (1 interview), operators of physical flea markets (3), online marketplace managers (2), and textile recyclers (1). These interviews enabled me to cross‐check many reseller statements and provided crucial context for the secondhand market. These additional interviews made for a total of 38 interviews.

Interviews occurred over Zoom and lasted 40–120 min, averaging 70. In interviews, I asked resellers questions such as ‘What was the first item you sold?’ and ‘How has your pricing practice changed since you started?’ to learn how resellers justified their pricing practices over time. I also included questions about their customer interactions, like ‘Can you recall a time when a customer doubted your price?’.

Using Atlas.ti, I initially coded the interviews according to my initial theoretical interests in valuation. Codes included ‘Perceptions of customers’, ‘Pricing changes’, and ‘Pricing disagreements’. Using Timmermans and Tavory's (2012) abductive analytic process, I compared my preliminary coding results to existing theories and developed new emerging codes such as ‘Invocation of a true market price’ and ‘Symbolic signifiers of value’.

I supplemented interviews with in‐person and digital observations. I spent 30 h in secondhand clothing sources and 42 h visiting 21 physical vintage markets in Chicago and Los Angeles. I produced field notes of my observations on resellers' presentations, prices, and customer interactions. In addition, I frequented online blogs and community boards where resellers conversed. I took digital photos of posts and wrote field notes on my perceptions and reflections. These posts ranged from personal selling celebrations to mundane complaints about marketplace structures. These ethnographic interactions allowed me to experience the materiality of reselling work, generate further questions for interviews, and document customer interactions.

### 3.1 The online secondhand marketplace environment

Marketplace boundaries are porous, as many sellers occupy multiple marketplaces in the same industry at once (Cutolo & Kenney, 2021, p. 23). Resellers primarily congregated in two marketplaces, Depop (20/31 resellers) and Etsy (14/31), but sold clothes in various venues, including the social media platform Instagram (15/31) and online marketplaces like Poshmark (9/31) and eBay (8/31). Each reseller used at least two different sites to sell, with some using up to seven.

Pricing formats differ by marketplace. Depop, Etsy, and eBay each require resellers to list a public price for an item without suggestive aids. Once resellers post items publicly online, they take offers on a rolling basis. Buyers purchase clothing for the stated fixed price or negotiate new prices using online chat features. Even on eBay, a site widely known for auctions, resellers preferred this negotiable format. The transaction completes after the reseller receives the payment and sends the item off. Instagram, primarily a social media site, is exceptional in this context. Resellers post prices and chat with buyers on Instagram without the transactional safeguards guaranteed by online marketplaces. Other popular marketplaces, like Poshmark and Mercari, use photo recognition software to offer pricing suggestions for listings based on matches with previous sales. Similarly, thredUp prices items using an algorithm after a reseller inputs item information online. However, algorithmic prices on thredUp are final, and the company completes listings automatically.

Despite differences in pricing formats, these marketplaces share similarities in their pricing displays and lack of authentication services. Both proposed and final prices and the listings are publicly available on most online marketplaces (but not Instagram, which does not primarily operate as a marketplace). Furthermore, these marketplaces do not have formal authentication services to aid resellers, and marketplace managers provide little instruction on verification.^5^ Resellers solely interpret items' age, condition, and cultural components.

## 4 FINDINGS

The findings section consists of two parts. The first part outlines the reselling process from searching, displaying, and choosing a marketplace. Each step highlights the valuation challenges that resellers face in preparing items for sale online and evinces several sources of pricing information. The second part examines resellers' pricing practices of resellers, focusing on the aesthetic and symbolic values they uphold and their interactions with marketplace technology. Then, I summarize how resellers selectively and recursively draw on communally shared values and technologies central to digital marketplaces to price secondhand clothing items.

### 4.1 The online reselling process

#### 4.1.1 Searching

Reselling begins when resellers search for items at physical secondhand sources, in the offline sphere. Within my sample, the two most discussed sources were thrift stores (used by 26/31 resellers) and ‘the bins’ (13/31), followed by estate sales (15/31), garage sales (7/31), and textile wholesalers (7/31). At these sites, resellers sort through thousands of clothing items from 1960s designer pieces to modern reproductions. In interviews, resellers mobilized many analogies to describe their search. They went ‘picking’ or ‘hunting’ to ‘strike gold’ or find ‘diamonds’. Despite romantic comparisons, Shelly, 21, from Southern Indiana, stated that she ‘learned a lot of patience… You can spend 3, 4, even 6, 7 h at one store and find two things… or nothing‬.’‬‬‬‬‬‬‬‬‬‬‬‬‬‬‬‬‬‬‬‬‬‬‬‬‬‬‬‬‬‬‬‬‬‬‬‬‬‬‬‬‬‬‬‬‬‬‬‬‬‬‬‬‬‬‬‬‬‬‬‬‬‬‬‬‬‬

At thrift stores (e.g., Goodwill and Salvation Army), the most popular source, employees receive bags of donated items from consumers. Workers sort through these bags, briefly inspecting items for cleanliness and necessity. This quick sorting permits the hundreds of items on sale in stores. They then price items using standardized scripts based on the type of clothing (pants vs. shirts: $6 for jeans and $4 for shirts at Goodwill) and general connotation of the brand (low‐tier Hanes vs. mid‐tier Banana Republic: shirts from $2 to $6 at Salvation Army). Thrift store workers price items at typically under $20 but usually only for a few, imprinting non‐negotiable prices on physical tags.

**FIGURE 1:**
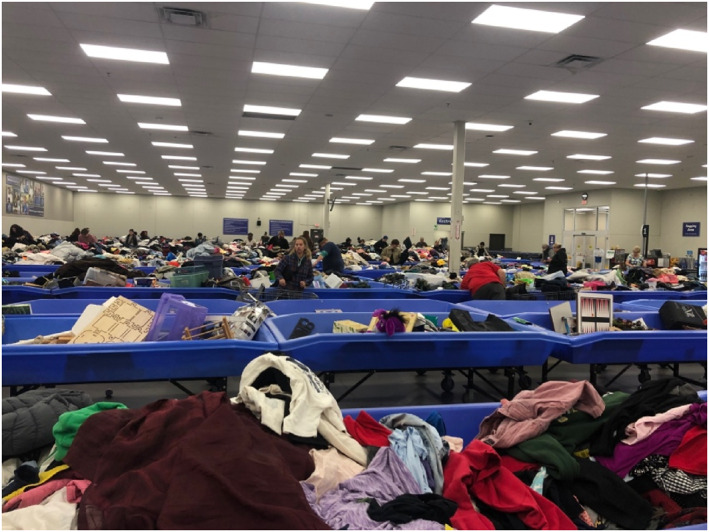
Inside ‘the bins’.

As for the bins or ‘outlets’, another popular source, Shelly explained: ‘After a certain amount of time of being in the main [thrift store] location, they'll send it all to the outlet stores … and it's priced … like $1.50 a pound.’ Resellers search through piles of clothing, which are periodically swapped out (Figure 1). Searching at the bins is ruthless and dirty (Ayres, 2017). Interviewees discussed norms governing proper searching, such as not crowding a bin when someone already has unofficially ‘claimed’ it by blocking physical space with their body, and many suggested using gloves to avoid filth.

Resellers look out for certain behaviors to identify colleagues at sources. ‘Most people, when they're shopping, are holding the item up to themselves, trying to see if they'll fit. Whereas … someone buying to resell it … they're just like throwing it in the cart,’ shared Lydia, 27, from Indiana. Trevor, 18, from Cleveland, shared he identified resellers: ‘When I see somebody with a shirt in their hand and they're looking at the shirt and they're looking back at their phone, shirt, phone, I'm like, “Okay, you're looking to see how much you could sell this for”.’ Still, resellers preferred working in isolation or with groups of pre‐existing acquaintances, rather than making connections in the field.

#### 4.1.2 Displaying

**FIGURE 2:**
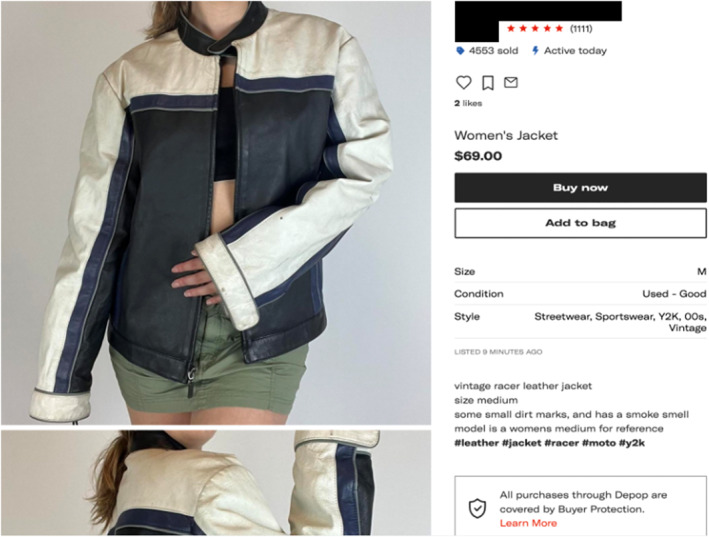
An example listing on Depop.

After purchase, resellers return to their place of business, which, besides a few successful operators, is their residence. There, resellers clean garments, take careful measurements, and photograph items. Resellers then upload content to individual item listings (Figure 2), writing captions and linking them to key search algorithms. They describe clothes using recognizable descriptors, sometimes creating word salads such as ‘vintage, cyber, y2k, Rockstar energy, AOP logo, print, faded, mall goth, emo, punk rock, t‐shirt‬’ (Bradley, 18, Florida).‬‬‬‬‬‬‬‬‬‬‬‬‬‬‬‬‬‬‬‬‬‬‬‬‬‬‬‬‬‬‬‬‬‬‬‬‬‬‬‬‬‬‬‬‬‬‬‬‬‬‬‬‬‬‬‬‬‬‬‬ These actions make items legible to the marketplace technologies and increase confidence in consumers who cannot physically inspect items (Kneese & Palm, 2020).‬‬‬‬‬‬‬‬‬‬‬‬‬‬‬‬‬‬‬‬‬‬‬‬‬‬‬‬‬‬

All the listings from one reseller on a specific marketplace collectively constitute a ‘shop’. Shops can contain up to hundreds of clothing items at one time. Shop names act as the brand name, and resellers produce style schemes that unite their operations on various marketplaces.

Resellers draw inspiration for their shops from others. ‘I was worried about how to post things… how to do shots. If I take a picture myself, is that enough? I had a hard time at first… ‬Popular sellers would do shoots… would have their page curated by colo‬r… So, I wanted to do something like that‬,’ remembered Chelsea, 25, from Chicago. Several resellers named high‐status resellers with popular profiles as inspiration (e.g., Depop seller Internetgirl and YouTube streamer Emma Chamberlain). By imitating high‐status actors, resellers attempt to imbue their wares with claims of high quality (Podolny, 1993, p. 831), improving their respectability (Aspers, 2009, p. 116) and ability to price items higher (Aspers, 2010, p. 49).‬‬‬‬‬‬‬‬‬‬‬‬‬‬‬‬‬‬‬‬‬‬‬‬

#### 4.1.3 Choosing a marketplace

Resellers carefully contemplate where to list specific items due to design differences and perceived userbases in online marketplaces.

Interviewed resellers mostly preferred marketplaces that granted them control over pricing like Depop, Etsy, and eBay. They also used Instagram for similar reasons. Yet Instagram's lack of structured interactions required first establishing a reputation online. ‘[You are] more successful if you get followers on Instagram… that's one of the benefits of having the [marketplace] platform… you pay their fee, but they provide a solid audience to put your product out to without having to market,’ shared Shelly. In contrast, resellers derided automatic pricing suggestions in marketplaces like Poshmark and thredUp that automated prices as clumsy valuation attempts. Nevertheless, they believed these marketplaces had their place. ‘We call it turn and burn, where you put it up for really cheap and you just sell it fast,’ shared Greg, 41, from Oregon, who used these sites to unload unwanted items.‬‬‬‬‬‬‬‬‬‬‬‬‬‬‬‬‬‬‬‬‬‬‬‬‬

Marketplace managers hold buyer information tightly, revealing only general demographic trends in promotional material. However, resellers still glean information about their buyers through structured interactions online. In interviews, resellers presumed buyers were predominantly Gen Z to Millennials (15–35 years old)–the digitally savvy–located in North America and Western Europe.

Resellers also establish boundaries with buyers. Many contested common perceptions of purchasing secondhand online as ‘thrifting,’ the colloquial term for bargain buying: ‘Somebody has already done the work of digging and curating stuff that's cool and desirable to entice you‬’ (Sara, 21, Tennessee). Though they often encountered exceptions, resellers believed most buyers held less interest and knowledge of clothes than they did.

This belief coincided with an apparent goal of these online marketplaces. According to one online marketplace expert:

There are the people who already do this… And on the other side of the spectrum is the skeptical secondhand shopper… folks who don't understand the value of secondhand. Those [two] are relatively small segments of the market… 10% each, I'd say … The opportunity is in the middle.‬‬‬‬‬‬‬‬‬‬‬‬‬‬‬‬‬‬‬‬‬‬‬‬‬

This middle population consists of those frustrated with the online, as opposed to the physical, shopping experience or those who want to shop secondhand but have no familiarity.

Alongside these broad generalizations, resellers believed different marketplaces attract different buyers and tried to match their pieces with an imagined audience. Tamara, 19, from Southern California said, ‘Depop seemed a little more focused… It's just clothing and so it seemed like a more viable option. eBay… just seemed a little bit sketchy.‬’ Bradley stated, ‘I've always wanted to try Etsy… Depop, their users don't appreciate true vintage… like seventies and before like, the old stuff, fifties workwear, stuff I like to collect‬.’ Evan, 25, from Chicago, provided an alternative: ‘I've heard good things about eBay. More people are on eBay… than they are on Depop. Depop is very catered to a younger audience, so I really wanna branch out… because that allows for a higher spectrum of age‬.‬’

After resellers decide on the proper marketplace, they publicize their prepared item listings. Resellers can then interact with potential buyers. Of course, when customers encounter a listing, they look at the price. In my interviews, resellers defined determining a suitable, legitimate as the core of reselling. While pricing concerns have appeared throughout my description of the reselling process, I dedicate the next section solely to pricing analysis.

### 4.2 The pricing practice of online resellers

Many factors influence pricing: for instance, status (Beckert, 2009, p. 118). Jim, 19, from Missouri, offered, ‘Not like they were just super low back then… but I was trying to get some sales under my belt, get views on my page, show other people that I'm credible. Once I gained credibility, I started raising the prices.’ Resellers claimed that being viewed as credible, through followings and completed sales, allowed them to increase prices. Yet, unlike in high fashion markets (Aspers, 2010, p. 49), status did not drive most reported price differences. All resellers had similarly small followings, from hundreds to thousands of followers. At most, some resellers stated that acquiring a following led to a general price increase of 15%.

Rather, resellers believed their experience in reselling interactions enabled them to determine prices. First, these interactions helped them appreciate key material, historical, and aesthetic item signifiers that increased an item's value. Resellers learn these signifiers from informal reseller networks and experiences at secondhand outlets. Simultaneously, they assess prices of comparable items using pricing displays on multiple platforms. Some resellers expressed a policy of price maximization and attempted to ‘squeeze’ out a piece's perceived market value derived from past market prices. While acknowledging this perceived market value, others were content with utilizing personally developed formulas and heuristics to secure profits, ensure the movement of goods, and maintain affordability.

These pricing elements–key signifiers and market price–often seemingly offer divergent price outcomes. However, most resellers said they blended their use of the two elements when justifying prices to customers. Resellers centered community values to support prices and then validated those prices with price displays on the existing online marketplace. Resellers thus critically justify and legitimate prices, produced by identifying community‐determined values, through the performative reflexivity of marketplace technology.

#### 4.2.1 The role of symbolic and aesthetic value signifiers

Resellers appraise the material and historical features of the clothes they sell through initial inspection and online research. Identification of such signifiers by resellers imbues a price claim with perceived legitimacy. While quality markers circulate in predigital secondhand contexts, like consignment stores and vintage boutiques (Fischer, 2015), digital culture determines altered value signifiers, including age, construction, brand, and aesthetics.

Since the original owner is not typically involved in pricing, resellers look for details to trace an item's history. Tags offer hints at an item's age. Lydia spoke about her process: ‘If you have an item that has an RN [Registered Identification] Number, you can look up the copyright of the company. It's not necessarily when the item was made, but it's when the company created that item and then first started using that pattern.‬’ Justin, 27, from the suburbs of Chicago, shared another quality signifier visible on tags: ‘Good old “Made in USA.”’ In interviews, resellers often referenced ‘Made in USA’ to denote a perceived quality of material and construction. Additionally, the phrase operated as a marker of historical authenticity, ensuring that clothing is genuinely vintage. Invoking the passage of NAFTA and globalization broadly, resellers signposted the 1990s as a period of transition from the mass production of predominantly American‐made clothing to non‐American production (Fischer, 2015).‬‬‬‬‬‬‬‬‬‬‬‬‬‬‬‬‬‬‬‬‬‬‬‬‬‬‬‬‬‬‬‬

**FIGURE 3:**
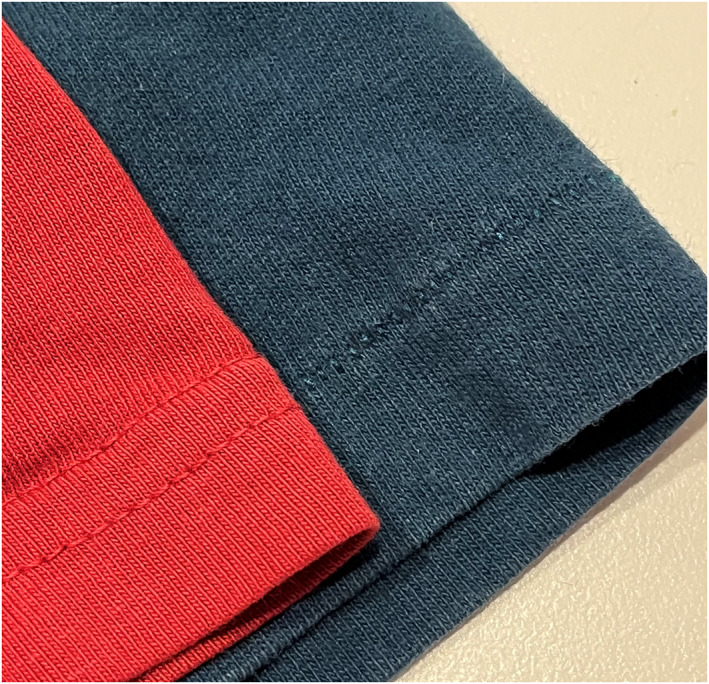
Comparison between a t‐shirt with a double stitch (left) and a single stitch (right).

Resellers can still read items without tags. T‐shirts feature a hem on the sleeves. ‘The old mills, they use[d] a type of machine that made the single stitch. Now science has made a faster, more efficient way to make a t‐shirt that uses that double stitch… So, kids are like, if it's single stitch, it was only made prior to 1996 or 1998,’ explained Owen, 21, from Florida (Figure 3).

Owen used these symbolic signifiers to identify possible valuable items: ‘Here are some shirts I got yesterday. It's like a Grateful Dead t‐shirt. And it checks off the boxes… from the collars, single stitch. It's tagged, the sleeves are single stitch and then it has a copyright… 1985. So, boom, this is a vintage^6^ T‐shirt….’

The brand is also an important signifier. Brands acts as quality markers in the general clothing market (Aspers, 2010, pp. 51–52; Beckert, 2020, p. 298). However, resellers assigned brands distinct meanings. Interviewed resellers prized brands that drove higher prices (e.g., Gunne Sax, a now‐defunct San Francisco‐based clothing company popular in the 1960s–90s that sold semiformal flowing dresses reminiscent of the Victorian era). Contemporary brands (e.g., Brandy Melville, an Italian‐based women's clothing brand synonymous with the modern California lifestyle) also attracted attention. On the other hand, resellers uniformly reviled commercial brands (e.g., fast fashion stores like H&M and Zara).

Fashion and style–‘aesthetic’ as my sample called it–play a role in pricing. Item selection approaches define a key division among resellers I spoke with. Regarding their presentation online, I categorized resellers as generalists (22/31 resellers) or specialists (9/31).

Some resellers consciously envisioned a look for their shops. ‘I want it to feel sort of a non‐defined alternative aesthetic … whether you're emo or goth or cottagecore or dark academia,^7^’ offered Jaime, 33, from Los Angeles.‬ They also focused on material aspects. ‘I really try to search for the higher end of the plus sizes, so anything that's 2X, 3X, and above,’ shared Chelsea.‬‬‬ I refer to these sellers as specialists as they used consistent descriptors to unite the items they sold and gained more knowledge on specific aesthetics to attract invested, niche buyers. Specialists often pass up items that do not fit into their brand and attempt to present a more coherent presentation.‬‬‬‬‬‬‬‬‬‬‬‬‬‬‬‬‬‬‬‬‬‬‬‬‬‬‬‬‬‬‬‬‬‬‬‬‬‬‬‬‬‬‬‬‬

Some resellers, whom I call generalists, adopted a more carefree attitude to styles, dabbling in many prevailing trends. Evan explained his collection of goods:

I sell what I find… I try to cater to what I think people would want, but in terms of like, do I only buy sports stuff, do I only buy horror movie promo stuff, like that? No, I buy and I pass on certain stuff, 'cause there's just certain stuff I don't wanna deal with, or like I know other people can sell better.

Resellers like Evan often recognized the possible value of items but believed they could not appropriately justify their price to customers. In these cases, they sold items to more or differently specialized resellers.

Resellers held strong opinions of their type and the other. Justification for a generalist approach typically revolved around avoiding missed profits. Each item might have some untapped values. On the other hand, specialists leveled criticisms that generalists adopt a ‘lazy’ attitude and perpetuate popular stereotypes of resellers as profit vultures, a common attack levied in discussions online. To themselves, specialists acted as interested taste‐builders, who work for the love of their craft and deserve the confidence of their buyers.

Resellers primarily learn of item characteristics, secondhand values, and aesthetic identification through informal connections physically and digitally. Owen learned by ‘just being around people… being the one that follows the older guys. It becomes common knowledge… So, I can't think back and be like, “this is the time I learned about it.”’ Only a handful of resellers mentioned working as an apprentice to a more experienced reseller, like Bradley: ‬‬‬‬‬‬‬‬‬‬‬‬‬‬‬‬‬‬‬‬‬‬‬‬‬‬‬‬‬‬‬‬‬‬‬‬‬‬‬‬‬‬‬‬‬‬‬‬‬‬‬‬‬‬

I met one bigger reseller at the Goodwill. A lot of other resellers will take you under their wing a little bit, teach you how to sell stuff. I have a friend who is 16 years old with over 500 sold who has taught me everything I know. So yeah, pretty much learning from other people…

Bradley then supplemented this experience with online sources, ‘the YouTube videos, I've tried those but those suck… Reddit is okay.’ So, instead of formal training, interviewed resellers acquired information through repeated interaction at the same local sites or the same digital forums.‬‬‬‬‬‬‬‬‬‬‬‬‬‬‬‬‬‬

#### 4.2.2 The role of market price

The digital marketplace infrastructure distinguishes online reselling from pre‐existing secondhand outlets. While discussions of symbolic and aesthetic signifiers provide critical insight, fully understanding reselling pricing practices requires consideration of online marketplace technologies. Here, the primary technological tool resellers use to form better estimations in relative isolation is online marketplace pricing displays.

Resellers reported using two pricing strategies: market ‘comps’ or formulas/heuristics. Fifteen resellers mentioned using only ‘comps,’ or comparison pricing, which originate from a synthesis of existing prices displayed on marketplaces.^8^ Five resellers mentioned solely using formulas or heuristics, based on labor, material, symbolic, and aesthetic inputs. Eleven stated they blended the two practices or used them at different times.

Finding a comp is simple but relies on the extensive price visibility essential to online marketplaces. Shelly explained:

I was searching for prices, and I would try to find the item or similar item on Depop and then price it competitively within what was currently listed… So, I would search for the item and then sort them low to high price… And then I would scroll through until I got to a point where I was like, ‘Okay, my item kind of fits at this level of quality.’

All resellers reported familiarity with comps, and many applied it to inspect secondhand items (as shared by Trevor on pg. 8). Some relied on other adjacent technologies to determine a comp. ‘There's this app called Gem, it's kind of like a Google for vintage. So, I usually go on there and type in a particular brand or style,’ shared Eva, 33, from Detroit. Using a search feature, the Gem app allows resellers to pull up related items and listed and sold prices from different marketplaces in one search, thereby streamlining the comp process.

Viewing online marketplace price displays through comps, resellers could grasp a purported ‘true’ market price. In this context, the comp acts as a prosthetic price (Çalışkan, 2009, p. 264), a socio‐technical tool resellers rely on to support their pricing claim. By invoking displays of settled prices to subsequently price their items at a ‘true’ market value, resellers reify the existence of a true market price. This practice then enables resellers to achieve higher prices than those charged at their charitable and fringe market secondhand sources.

Other resellers used different pricing tools. Pricing formulas or heuristics incorporate a multiplication of an item's purchase price to cover labor costs, item elements, and some market value. Justin provided his method:

There are people who are trying to get full‐blown value … they look at [the] ‘comp’… Then there's me, who is like, ‘Okay, I paid 5 bucks for this hat. I got five bucks in it to restore it. It's a cool hat … 30 bucks.’ So, there are certain things that in my head have certain prices already.

Jaime broadly applied a different formula:

I do the math for the profit margin to come out right… How much is this gonna cost to ship? What's the sizing on it? … Is it in line with my shop or will sell on Depop?‬ … It's a formula that's always adapting every few weeks, given the feedback loop of what's going on Depop versus where am I at financially versus how cute is the piece. It's just like a formula that constantly sort of evolves‬… But it, for me at least, does have a lot to do with profit margin, shipping‬, etc.‬‬‬‬‬‬‬‬‬‬‬‬‬‬‬‬‬‬‬‬‬‬‬‬‬‬‬‬‬‬‬‬‬‬‬‬‬‬‬‬‬‬‬‬‬‬‬‬‬‬‬‬‬‬‬‬‬‬‬‬‬‬‬‬‬‬‬‬‬‬‬‬‬‬‬‬‬‬‬‬‬‬‬‬‬‬‬‬‬‬‬‬‬‬‬‬‬‬‬‬‬‬‬‬‬‬‬‬‬‬‬‬‬‬‬‬‬‬‬‬‬‬‬‬‬‬‬‬‬‬‬‬‬‬‬‬‬‬‬‬‬‬‬‬‬‬‬‬‬‬‬‬‬‬‬‬‬‬‬‬‬‬‬‬‬‬

Those who preferred or exclusively used formulas contrasted their approach to the ‘hungry mercenary attitude’ (Andrea, 38, Chicago) of resellers who practiced profit ‘maximization’. Instead, they prioritized affordability and simplicity.

Contrary to the presumed belief that resellers who adopted a generalist approach were money‐hungry, there appeared to be no connection between the two stylistic types (generalists and specialists) and the use of price maximization or formulas. Each stylistic type was just as likely to adopt formulas, comps, or blend the two. Therefore, differences in interest in fashion or status within a particular aesthetic niche do not explain differences in pricing practices.

#### 4.2.3 Unified pricing practices

Like with their approaches to stylistic visions, resellers hotly debate the relative importance of signifiers and market prices in pricing within their community. Yet, the importance of both is evident in their varied pricing practices.

Successfully applying a comp requires a deft eye for item signifiers, sharpened through trial and error. Almost all interviewed resellers, such as Owen, regretted missing details in previous sales:

The first thrift I sold … was a Harley Davidson shirt. It was cool because it's called a 3D emblem, and they're like made in the eighties… I remember I pulled it, and I had no clue what it was. It's like a $200, $300 shirt. I sold it for like $70… People bought it so fast because someone knew what it was, and they probably flipped it. But it was cool being like, ‘Whoa, it's worth something.’

Lydia shuddered speaking about how her assessment process had evolved:

I learned how to differentiate between things that have true value versus things that you can price however. I sold quite a few rare pieces for dollar amounts that hurt me to think about… I’m thinking of this seventies Made in California Beach Boys Tyvek jacket that they sold on a tour… It was so cool, and I sold it for probably… like $45 or something.

Prices can also appear illegitimate when resellers misinterpret specific signifiers that reveal more accurate comps. That failure draws contestation from buyers and other resellers, invalidating a stated price. An adamant user of comps, Kathryn, 52, from Indianapolis, recalled one such instance:

Often you can find the same item that somebody's asking $20 for and somebody else is asking $200… Occasionally I've been wrong … I had a Spirited Away [popular Japanese anime] t‐shirt… Somebody was asking like $200, but [mine] didn't have a tag. So initially, I was like, ‘Well, I know people really love anime, so maybe I'll just get [$20].’ And then I was like, ‘Oh, wow, it says 2001 on the printing inside,’ which is the copyright date, but I thought perhaps that was the actual manufacturing date. And so, I think I asked like $100 for it. And then somebody just the other day was like, ‘Girl, you can get that for $15 at Hot Topic.‬‬’‬‬‬‬‬‬‬‬‬‬‬‬‬‬‬‬‬‬‬‬‬‬‬‬‬‬‬‬‬‬‬‬‬‬‬‬‬‬‬‬‬‬‬‬‬‬‬‬‬‬‬‬‬‬‬‬‬‬‬‬‬‬‬‬‬‬‬‬‬‬‬‬‬‬‬‬‬‬‬‬‬‬‬‬‬

Alternatively, resellers who preferred the use of formulas and heuristics, like Spencer, 27, from Arkansas, struggled to make sense of high marketplace prices:

This guy sold a 1992 Aladdin [movie promo] t‐shirt… for $6,000 on Instagram… Now you have people who think their stuff might be a lot more valuable. But one sale doesn't necessarily dictate the market value… It kind of puts us all in this weird impasse where you price things at what you think people are willing to pay. But you don't want to neglect the data out there that someone did pay $6,000 for that t‐shirt. I feel like a sucker for getting anything less. ‬‬‬‬‬‬‬‬‬‬‬‬‬‬‬‬‬‬‬‬‬‬‬‬‬‬‬‬‬‬‬‬‬‬‬‬‬‬‬‬‬‬‬‬‬‬

Spencer needed to negotiate market hype, which he defined as wildly fluctuating prices due to ephemeral demand, with underlying value, which he tied more directly to signifiers. Regardless of his emotions regarding hype, Spencer felt compelled to capture some value of market pricing into his valuation or else be a ‘sucker’.

Justin, another adamant comp denier, similarly rejected the high prices dictated by ‘true market value’, but with caveats:

We're trying to give it a new home at a good price, and not like $100 for a pair of Levi's jeans. You gotta be out of your fucking mind … unless they're like old as shit…

In almost all interviews, resellers repeated this internal dispute over the appropriateness of a floating market price and the fear of misrecognizing the online market demand. This conflict reflects their struggle to identify knowledge that could legitimate their prices.

## 5 DISCUSSION

Resellers employed valuation approaches that respected key item signifiers while granting marketplace displays, and their fluctuating signals, considerable influence. Resellers therefore do ‘performative work’ at the micro‐level to integrate new market devices into the secondhand clothing market, while respecting the market's particular ‘organizational, institutional and political context’ (Beunza & Ferraro, 2019, p. 516). Collectively, these pricing practices, which acknowledge both signifiers and market price, resemble a process of ‘recursive pricing’ in which ‘prices function as engines of interaction in the recursive, mutual constitution of… the coordinating social structures that shape prices and… the performance of’ a standard of truth ‘that realize prices’ (Bååth, 2023, p. 18).

Why do resellers utilize such pricing practices?

Online marketplaces exacerbate price justification challenges already present in symbolic markets (Boltanski & Esquerre, 2020). Popular marketplaces rely on the influx of users to drive scalable profits. Their infrastructures make rapid entry easy for sellers and buyers. However, most online secondhand marketplaces do not employ costly verification services to keep costs down, and no outside institutions exist to support resellers' pricing claims on items. Therefore, resellers encounter varying levels of expertise and knowledge in customers and must quell criticism and inspire confidence without the aid of critics, experts, or other signs of legitimacy.

Resellers frequently recalled receiving angry and skeptical messages about their prices. Sophia, 25, from Los Angeles, talked about those on the outskirts of the market:

[They’re] not your clients… Go find a fucking cargo pants for 10 bucks! They don't know that [vintage]'s going to last them way longer than when they go every Saturday to the mall to get some shit from Zara or H&M… that’s going to be gone in two weeks…‬‬‬‬‬‬‬‬‬‬‬‬‬‬‬‬‬‬‬‬‬‬‬‬‬‬‬‬‬‬‬‬‬

Dominic, 64, from Chicago, shared this frustration: ‘It goes back to this expression: An educated consumer is the best consumer… because they know what they're looking at. If you have to teach someone what they're looking at, they're not going to buy it‬.’ ‬‬‬‬‬‬‬‬‬‬‬‬‬‬‬‬‬‬‬‬‬‬‬‬‬‬‬‬‬‬‬

Yet, knowledge of secondhand is a spectrum. Bradley ruminated on the diversity of buyers:

There are buyers who will say nothing and then they'll buy a nice item. … Then there are buyers‬‬‬ like… this happens all the time. I have this all‐overprint 2000s shirt listed for 25 bucks by ‬‬‬Liquid Blue going on eBay for 45 bucks and one for 60 bucks. This dude, probably 14 years old, messaged me and says, ‘Best offer, boss?’ I honestly have it priced low, but I could go down to 20 plus five shipping for you. ‘Can we do 15?’ ‘I'm sorry. Someone else already has one listed at 45. It's already a steal. Hope you can understand.’‬‬‬‬‬‬‬‬‬‬‬‬‬‬‬‬‬‬‬‬‬‬‬‬‬‬‬

Legitimating these prices relies on recursive practices. In person and on social media, resellers learn information on communally valued clothing elements from fellow resellers, and then they utilize these cultural values to respond to criticism. To many buyers, demonstrating these values suffice, but they are often insufficient for the ‘uninitiated’ casual ones (Wherry, 2008). More justification is needed. Under questioning from buyers, resellers connect their items to past interactions on online marketplaces, primarily through the free‐floating comp and reactive feedback calculations. After all, buyers can access many of the same tools available to resellers since all prices are available and searchable, providing support for valuations if contested. While some resellers resist faithful privileging of the ‘true market price’, its power is undeniable for most. This integrated approach to pricing analysis thus elucidates price agreements in this online context which contains few traditional institutional guarantees or approvals.

Determining salient item qualities and pinning a price makes unique, non‐standard secondhand clothing items sellable. This achievement mirrors that of qualification, introduced by Callon et al. (2002), which refers to the ‘controversial processes… through which qualities are attributed, stabilized, objectified and arranged’ (Callon, Méadel, & Rabeharisoa, 2002, p. 199). In the secondhand case, sellers and buyers define the objects for sale and classify them appropriately. As a result, market actors determine an item's semi‐singularity but also similarity, allowing buyers to make rational choices while mobilizing qualified, symbolic elements (Cochoy, 2008, p. 30). So, by establishing objectified qualities of clothing and attributing suitable prices in transactions through recursive pricing, resellers succeed in establishing secondhand clothes, long relegated as cheap, charitable items, as items suited for market trade.

Overall, introducing online marketplaces into the traditionally offline secondhand clothing market has provided new challenges but new tools for reselling. While beyond the formal scope of this analysis, online marketplaces appear to enable secondhand clothing trade expansion. By removing many geographic challenges to physical trade, online marketplaces expand a possible customer base for shops. While increased users of differing knowledge and experience pose challenges to trade, by filling these marketplaces with interactions, resellers can observe considerable pricing information to determine and justify relatively higher prices compared to past secondhand formats. My interviews with primarily offline resellers revealed the pervasive influence of the online marketplace displays in the general secondhand market. Yet, appropriate use of these new tools requires deft application, and resellers must carefully attend to community structures and marketplace interactions to profit.

## 6 CONCLUSION

How pricing operates on online marketplaces–a unique market form–is undertheorized (Aspers & Darr, 2022). This article examines the pricing practices of secondhand clothes resellers: participants in a market environment where several prominent online marketplaces have emerged. Resellers price symbolic and irregular material items for sale online while lacking formal ties and operating across a wide physical geography. Furthermore, these online marketplaces expand popular participation. While instituting a variety of technological features to regulate behavior and limit concerns, these online marketplaces do not possess market institutions that provide guarantees of pricing claims. All considered, resellers operate in an uncertain, heavily technological market environment. So, this case offers a theoretically compelling opportunity to analyze pricing on online marketplaces.

I apply recently developed unified pricing theories to analyze resellers' pricing practices and demonstrate how they converge. I show how reselling community structures and the performance of market value shape pricing practices. I theorize that online sellers respond to legitimacy challenges stemming from popular participation in online marketplaces using these pricing practices. As a result, this article adds to the sociological literature by utilizing novel pricing theories in a novel context and revealing how marketplace participants overcome unique online marketplace challenges.

Several underexplored themes in this article provide future pathways for research. The first theme concerns within‐industry marketplace variation (Rilinger, 2024, p. 2). Resellers encountered different pricing formats, from user‐generated to automatic pricing. Users occupying a particular trade area often use several marketplaces simultaneously to take advantage of competing offers through a strategy called multi‐homing (Cutolo & Kenney, 2021, p. 23). While this analysis mentioned perceptions of these differences, further research on how sellers assess and navigate these varying formats can provide insights into how marketplaces are generally ordered online. Another theme relates to various selling positions that resellers assume. Some resellers take on different roles in the marketplaces at different times. For instance, certain resellers purchase items wholesale and offload them to others, compared to the typical direct‐to‐consumer focus. The organic specialization of heterogeneous roles may also reveal how the community of resellers responds to the online marketplace environment.

Still, this study helps elucidate key processes of secondhand clothing markets and other market cases for symbolic, singular, and vintage material items (Boltanski & Esquerre, 2020). Secondhand clothing markets have long existed in the US, primarily in charitable and fringe market contexts (Le Zotte, 2017). While a healthy global trade exists where secondhand clothing is a commodity (Norris, 2012), the emergence of online marketplaces has ushered in rapid marketization and market growth (thredUP, 2024). Various symbolic industries have also quickly adapted to online marketplace formats, like vinyl records (Kneese & Palm, 2020), sneakers (Choi & Lee, 2021), and art (Fernandes & Afonso, 2020). As in the secondhand case, these online market environments experience similar conditions of uncertainty. Unified pricing theories, which respect mixtures of similar technological and social structural elements, likely serve as effective tools for detailing market orders in these cases.

## CONFLICT OF INTEREST STATEMENT

The author declares that he has no conflict of interest.

## ETHICS STATEMENT

The author received approval from their university's Institutional Review Board

## Data Availability

The data supporting this study's findings are available on request from the corresponding author. The data are not publicly available due to privacy and ethical restrictions.

## ENDNOTES

^1^
Only in vintage boutiques and consignments, which often focus on high‐end clothing, do secondhand items receive non‐bargain prices offline (Fischer, 2015).

^2^
By the end of 2024, industry experts predict that secondhand sales will constitute 10% of the global clothing market (thredUP, 2024).

^3^
‘Resellers’ refers to people who buy and resell secondhand clothes online. ‘Sellers’ refers to those who sell products on marketplaces generally.

^4^
Secondhand marketplaces typically cloak their activities in sustainability rhetoric. While many resellers expressed sustainability rhetoric as motivation for their work, most cited early profit as the primary reason for continuing to resell.

^5^
Unlike sites that focus on ready‐to‐wear clothes, high‐end clothing sites, such as the RealReal and Grailed, offer machine learning and online human review authentication services for some items.

^6^
Resellers utilize different terms to refer to their items. Despite disagreement, ‘vintage’ typically demarcates clothing at least 20 years of age. ‘Secondhand’ is all‐encompassing and includes vintage and modern pieces.

^7^
‘Emo’ and ‘goth’ refer to dark, haunting clothing associated with their respective musical genres. ‘Cottagecore’ evokes nostalgic depictions of pastoral life through lace and knits. ‘Dark academia’ draws on gothic European themes with richly colored scarves and cardigans.

^8^
Degenshein (2017) documented how pawnbrokers use online marketplaces to generate comps when negotiating with customers on the terms of loans backed by objects as collateral.
